# A Rare Case of Spontaneous Elbow Osteomyelitis Presenting With Aseptic Effusion

**DOI:** 10.7759/cureus.80305

**Published:** 2025-03-09

**Authors:** Eddie G Rodriguez Aquino, Miguel F Agrait Gonzalez, Sarah Marrero Medina, Israel Laracuente

**Affiliations:** 1 Emergency Medicine, Centro Medico Episcopal San Lucas, Ponce, PRI

**Keywords:** arthrocentesis, elbow effusion, elbow ultrasound, osteomyelitis, septic arthritis

## Abstract

Spontaneous osteomyelitis of the capitellum is an exceptionally rare condition, particularly in the absence of identifiable risk factors or inciting events. This case report describes a previously healthy 20-year-old male who presented with progressive elbow pain and swelling, initially raising suspicion for septic arthritis. Point-of-care ultrasound (POCUS) identified a significant joint effusion, and subsequent arthrocentesis revealed purulent fluid with a high white blood cell count, strongly suggestive of a septic joint. The patient was treated empirically with antibiotics and underwent surgical debridement, but cultures from both the arthrocentesis and the surgical washout were negative. Further imaging with MRI ultimately revealed findings consistent with osteomyelitis of the capitellum. This case highlights the diagnostic challenges associated with musculoskeletal infections, particularly in atypical sites like the capitellum, and underscores the importance of advanced imaging modalities such as MRI when initial diagnostics are inconclusive. The report also emphasizes the utility of POCUS in identifying joint effusions and guiding arthrocentesis in the emergency setting. Early recognition and appropriate management of osteomyelitis are crucial to avoid serious complications such as chronic infection, joint dysfunction, or limb deformity. This case contributes to the limited literature on capitellum osteomyelitis, particularly in the setting of an aseptic effusion, and advocates for the integration of multidisciplinary approaches and advanced diagnostic tools in the evaluation of pediatric and young adult musculoskeletal infections. Further research is needed to better understand the pathophysiology and management of rare presentations like this one.

## Introduction

Osteomyelitis is defined as an infection of the bone whose pathophysiology can range from an acute to a chronic process and is characterized by inflammation of the bone and bone marrow. The cause of the inflammation is due to a bacterial infection that occurs via different mechanisms such as hematogenous spread, direct inoculation from trauma or surgery, or contiguous spread from adjacent soft tissue infections [1]. In pediatric patients, the epidemiology of osteomyelitis is characterized by a variable incidence, estimated to range from two to 20 cases per 100,000 children annually [2]. Of all the bacteria that could cause this pathology, *Staphylococcus aureus* is the most common causative organism [3]. 

Osteomyelitis most commonly affects long bones such as the femur or tibia, but its presence in bony structures that compose the joints is rare. One such rare location is the capitellum [2]. Data for osteomyelitis of the capitellum is scarce, in particular for spontaneous osteomyelitis, without an obvious source or inciting event. Osteomyelitis may lead to significant complications, such as limb length discrepancies and deformity, pathologic fractures, subperiosteal abscesses [4,5], and even some cardiopulmonary complications [6]. A common complication in this population is septic arthritis of the joint [7] due to contiguous spread that occurs from transphyseal vessels that allow for the spread of infection from the metaphysis to the epiphysis and then into the adjacent joint, leading to septic arthritis [2]. The diagnosis of septic arthritis is suggested via the presence of synovial fluid that shows elevated total white blood cell count (WBC >50k), predominance of polymorphonuclear neutrophils (PMNs >90%), a positive Gram stain, or a fluid culture positive for bacteria [8]. 

We present a case of a patient with spontaneous osteomyelitis of the capitellum who presented to the emergency department with findings suggestive of a septic arthritis of the elbow. Both laboratory and operative findings were initially suggestive of a septic effusion, with no obvious infectious source identified. The patient was managed initially as having a septic joint; however, MRI eventually revealed osteomyelitis of the capitellum as the source of the effusion and elbow pain. The effusion itself was ultimately found to be aseptic and inflammatory in nature as a result of the osteomyelitis. This case highlights the importance of recognizing osteomyelitis as a potential cause of effusion, as management will change based on this diagnosis.

## Case presentation

**Figure 1:**
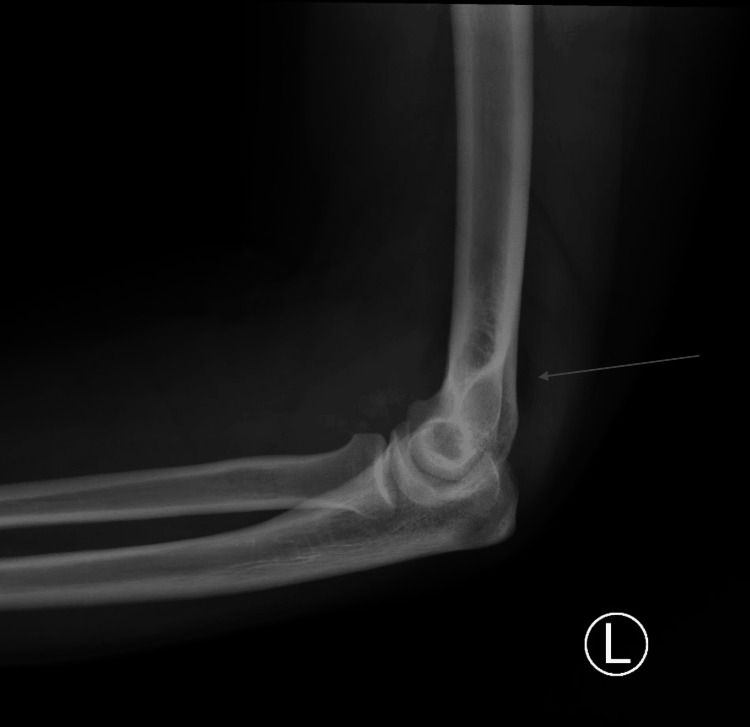
X-ray of the left elbow with posterior fat pad. The arrow indicates the posterior fat pad consistent with elbow effusion.

A 20-year-old male with no past medical history presented to the emergency department complaining of left elbow pain. The patient stated that symptoms started four days prior to evaluation without any obvious inciting event. Specifically, he denied any trauma, insect bites, breaks in the skin, sports injuries, injection drug use, recent dental procedures, or multiple sexual partners. The patient acknowledged that the left elbow had become progressively more swollen over the preceding four days. The day of the evaluation, the patient noted he was unable to fully extend his elbow, keeping it flexed to avoid discomfort. He denied fever, headache, sore throat, chest pain, cough, abdominal pain, nausea, vomiting, diarrhea, neurological symptoms, joint erythema, joint discharge, myalgias, tooth pain, or any medical or dental procedures in the past few weeks. On examination, the left elbow joint showed moderate swelling, decreased range of motion (ROM) in particular with regards to extension, painful ROM with active motion, painful passive ROM, and tenderness on palpation of the lateral elbow and the lateral proximal forearm without the presence of erythema or skin changes. On both upper extremities, the patient showed a normal capillary refill (<2 seconds) and intact sensation. An X-ray of the left elbow (Figure 1) was ordered, and it showed evidence of a posterior fat pad suggesting intra-articular effusion or occult fracture. In the absence of trauma, fracture was thought to be an unlikely diagnosis; therefore, the decision was made to perform a bedside ultrasound to better assess the joint.

**Figure 2:**
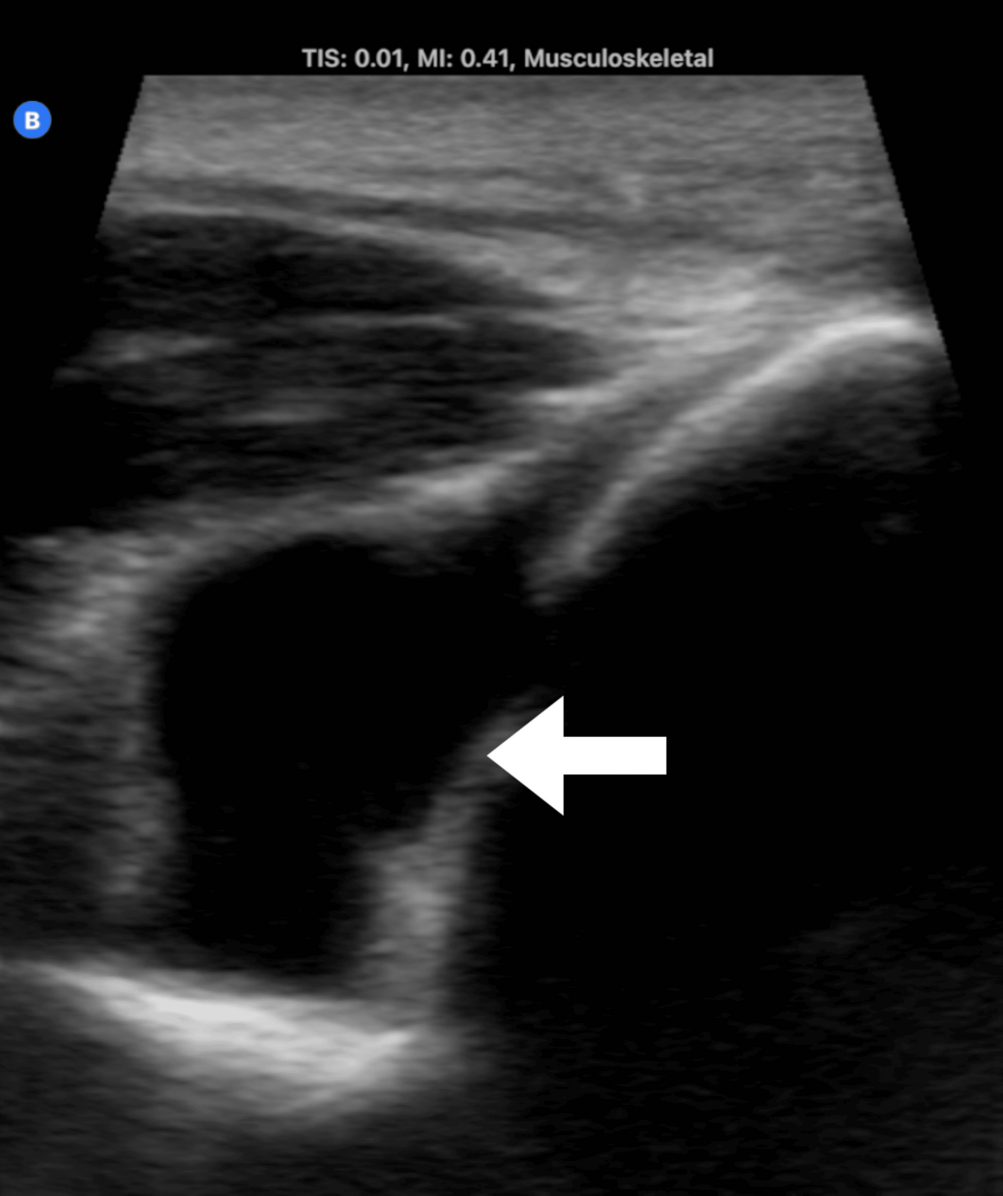
Effusion identified in the left elbow via ultrasound. A longitudinal view of the joint reveals an anechoic fluid collection. The arrow indicates the location of the anechoic effusion in the left elbow joint.

**Figure 3:**
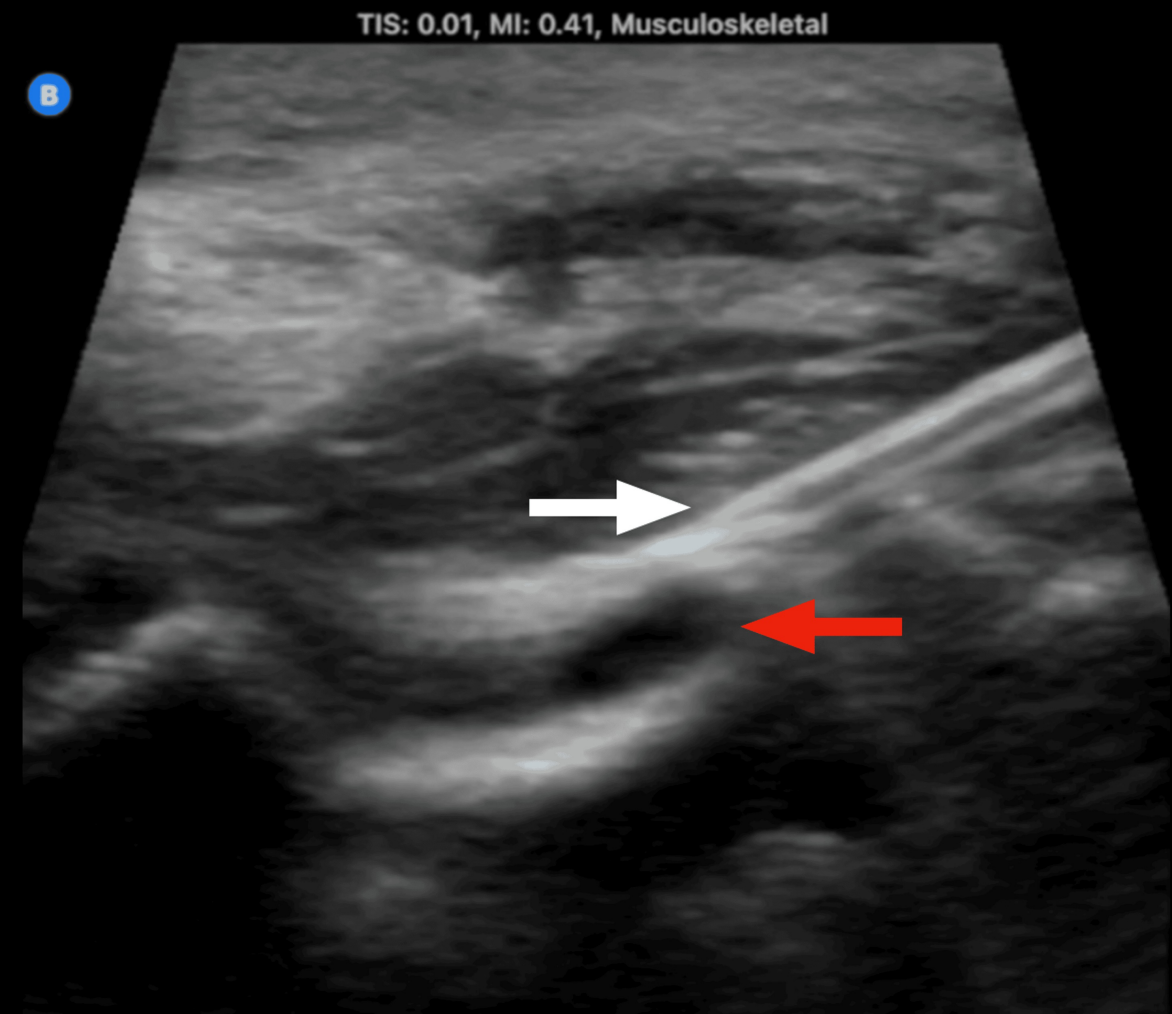
Ultrasound of the left elbow during arthrocentesis of the joint, with some residual effusion at the joint space. The white arrow indicates the needle, while the red arrow indicates joint effusion.

**Figure 4:**
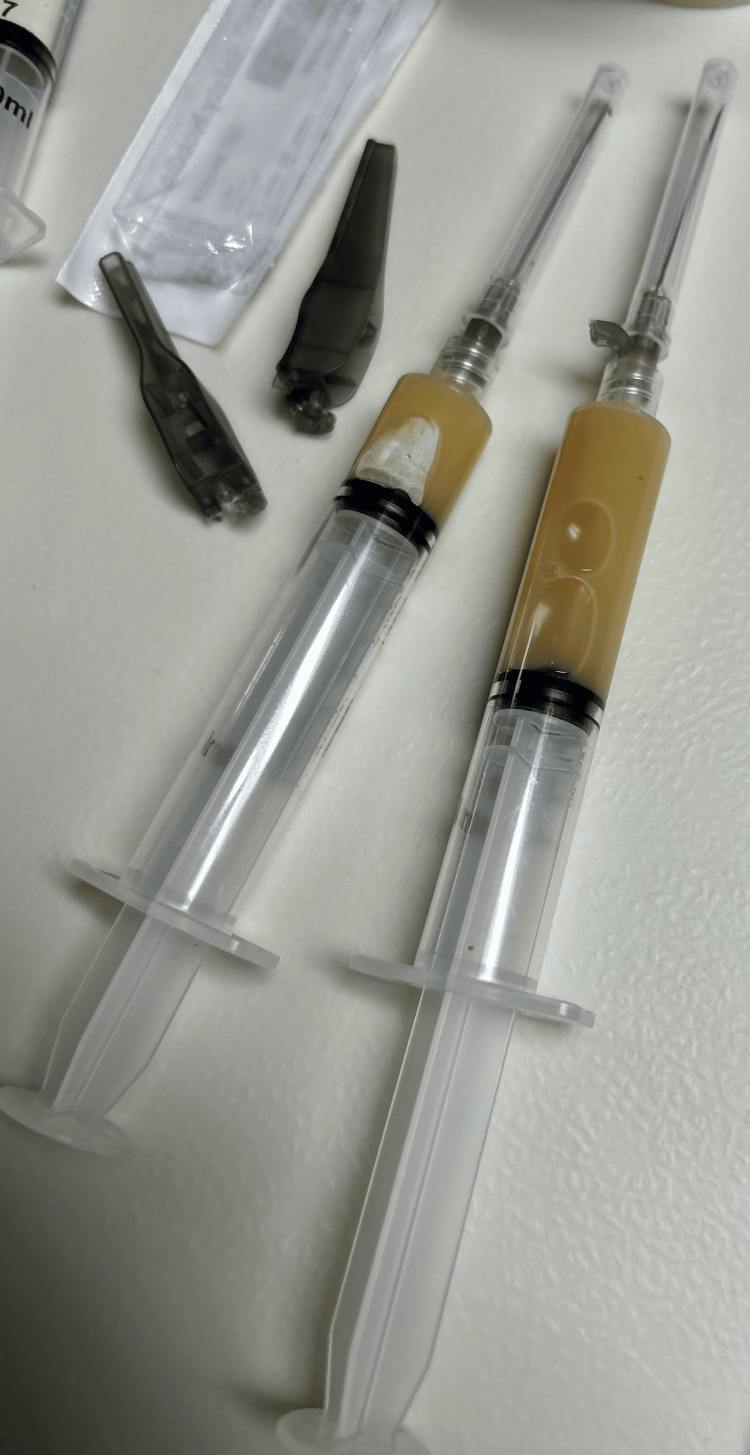
Fluid drained from the left elbow.

Using ultrasound, an intrarticular elbow effusion was identified (Figure 2). After obtaining the patient’s consent, an ultrasound-guided arthrocentesis of the elbow was performed (Figure 3) that showed a yellow opaque appearance most consistent with purulent drainage (Figure 4). Initial synovial fluid results showed the presence of WBC at almost 129,000, with more than 95% PMNs in the effusion (Table 1). Synovial fluid results were concerning for septic arthritis of the joint, so the patient was started on vancomycin and cefotaxime. The patient was transferred to a tertiary care center for further orthopedic care.

**Table 1:** Left elbow synovial fluid results. WBC: White blood cell count; PMNs: polymorphonuclear neutrophils

Synovial Fluid Analysis	Value	Reference Range
Synovial color	Yellow	Clear
Synovial appearance	Cloudy	Transparent
Synovial WBC	129,037 cells/mm³	<200 cells/mm³
Synovial PMNs	95.2%	<25%
Synovial Gram stain and culture	No growth at 72 hours and at 5 days	No growth

**Figure 5:**
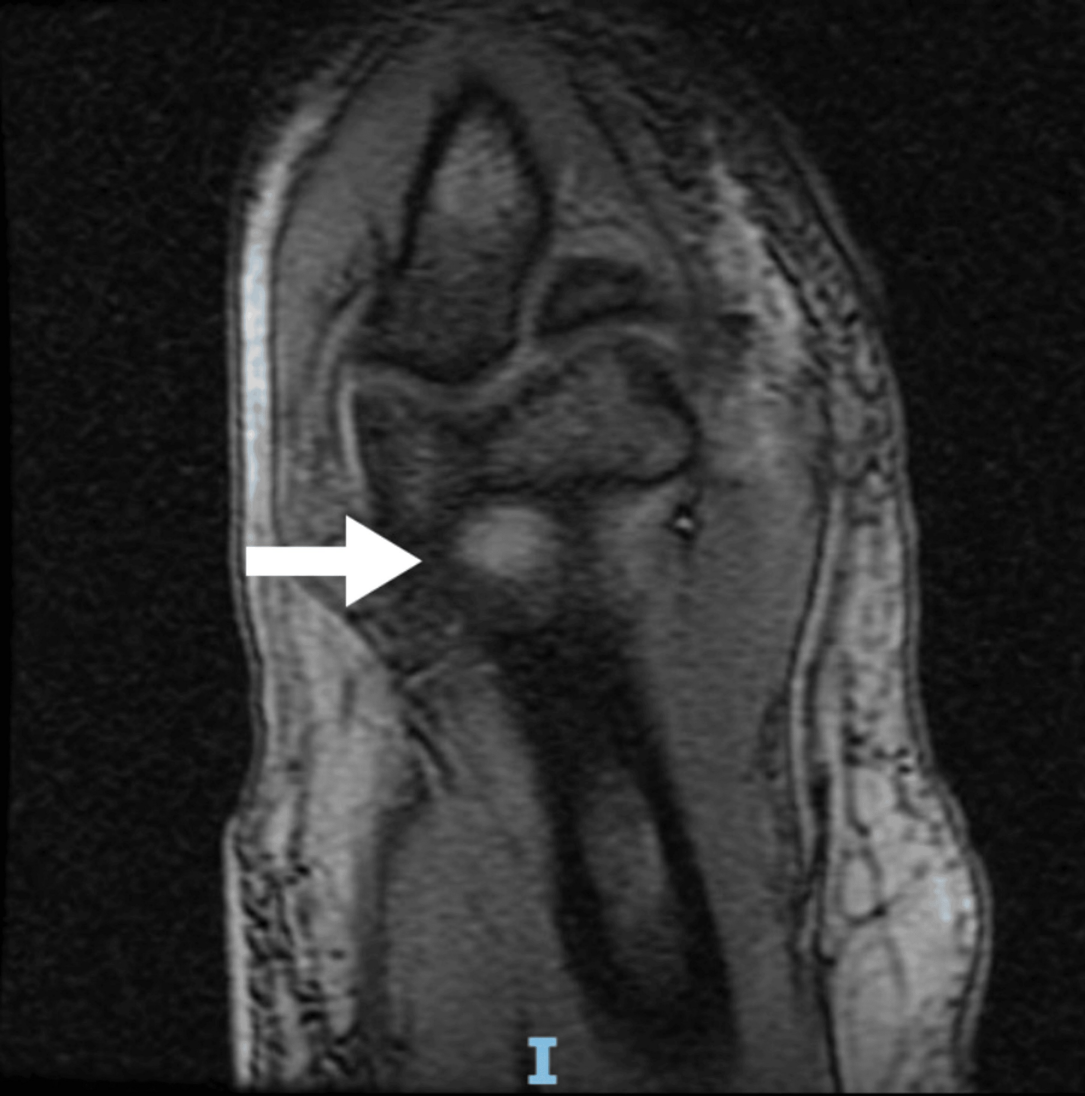
Left elbow MRI. The arrow indicates the location of the capitellum with bone marrow edema and changes suggestive of osteomyelitis.

Upon arrival at the accepting institution, the patient was admitted to the orthopedics service for surgical debridement of the affected joint. Operative report showed that the drained fluid was purulent in nature and concerning for septic arthritis of the left elbow. After five days, the wound cultures from both the operative procedure and arthrocentesis of the patient were finalized and showed no growth (Table 1). Sexually transmitted infection testing was also performed, and it was similarly negative in synovial, urine, and genital samples. At that time, the orthopedics team decided to perform an MRI of the affected joint, which showed findings consistent with osteomyelitis of the left capitellum (Figure 5). The infectious disease department was consulted, who evaluated the patient and concurred with the diagnosis of spontaneous osteomyelitis of the left capitellum. The recommended treatment was a total course of six weeks of IV antibiotics. After two weeks, the patient was discharged home where he continued his course of antibiotics and had an uncomplicated treatment course.

## Discussion

Spontaneous osteomyelitis is a rare occurrence, particularly in a previously healthy patient without any apparent risk factors. When present, it is most often acute and usually the result of hematogenous spread [9]. According to Goergens et al., findings of osteomyelitis can be subtle, and typical inflammatory changes such as warmth, swelling, erythema, and limited ROM are only seen in about 50% of cases. They identified that a normal corporal temperature, WBC, or erythrocyte sedimentation rate (ESR)/C-reactive protein (CRP) did not exclude the diagnosis of either osteomyelitis or septic arthritis [10]. Therefore, a high suspicion is warranted for both osteomyelitis and septic arthritis, as both conditions can lead to significant morbidity and potentially mortality if not diagnosed and treated promptly. In this case, physical exam findings of an unexplained effusion led to an evaluation for septic arthritis, and although the final diagnosis was ultimately an alternate one, the evaluation and management for presumed septic arthritis was warranted. 

A retrospective review by Schallert et al. attempted to determine the incidence of concomitant septic arthritis in the setting of metaphyseal osteomyelitis in pediatric patients. The study reported coexisting septic arthritis in 74% of cases with metaphyseal osteomyelitis and associated epiphyseal effusion. They concluded that in the setting of metaphyseal osteomyelitis, a joint effusion identified on MRI should be presumed septic until proven otherwise. Other MRI findings may be suggestive but are not as reliable as joint effusion in predicting septic arthritis [11]. In another retrospective study looking at the frequency of coexisting osteomyelitis in children with septic arthritis, Monsalve et al. reported osteomyelitis in 68% of cases with diagnosed septic arthritis. They found this to occur most commonly in older patients, while isolated septic arthritis was more frequently reported in children under two years of age. The study highlighted that strictly adhering to guidelines commonly used for the management of septic arthritis might lead to potentially missing cases of underlying osteomyelitis. In their conclusion, the authors advocated for the inclusion of MRI in the imaging workup of suspected septic arthritis [7]. These studies not only emphasize the potentially high rate of simultaneous incidence of two serious musculoskeletal infections but also promote MRI as a key feature in the diagnosis of osteomyelitis. 

A review by Pugmire et al. described radiography as a useful first-line imaging modality for excluding other differential diagnoses but underlined that it may miss other changes suggestive of early osteomyelitis and perhaps even the extent of disease as opposed to the high sensitivity of MRI for both [12]. Merlini et al., in a retrospective review, looked at concomitant septic arthritis and osteomyelitis of the hip in young children. The authors challenged the traditional understanding of how septic arthritis and acute hematogenous osteomyelitis develop, proposing that septic arthritis can precede osteomyelitis. They argued that MRI can pick up subtle findings, specifically decreased perfusion of the femoral epiphysis, that could be an early indicator of impending metaphyseal osteomyelitis [13].

Our patient presented a rare finding of osteomyelitis in the setting of an inflammatory joint effusion. Missing out on this diagnosis could potentially be deadly and/or a source of significant comorbidities. In children, delayed treatment of osteomyelitis can cause limb length discrepancies, limited ROM of the affected extremity, and persistent difficulties with weight bearing [14], while in adults, it can lead to chronic pain, early arthritis, recurrent infections, pathologic fracture, among other potential complications. The use of ultrasound was very helpful in the early diagnosis of our patient’s elbow effusion, which, in the absence of trauma, led to quick suspicion of septic arthritis and subsequent arthrocentesis. As described, the patient underwent surgical irrigation and debridement of the joint, but ultimately, follow-up MRI led to the diagnosis of spontaneous capitellum osteomyelitis in the context of negative synovial fluid and blood cultures. With all of this in mind, we support the addition of MRI in the workup of confirmed septic arthritis. 

This case also highlights the utility of point-of-care ultrasound (POCUS) in the diagnosis and management of patients in the emergency room presenting with articular pain. The presence of an unexplained effusion should prompt consideration of an arthrocentesis to evaluate for a possible septic joint. No lab or physical examination findings are sensitive enough to rule out a septic joint without an arthrocentesis. Therefore, this is a procedure that emergency medicine specialists should feel comfortable performing. In the case of suspected septic arthritis of an unusual joint, such as the elbow, if the treating physician does not feel comfortable performing the diagnostic procedure, early orthopedic consultation should be considered to establish the diagnosis of the septic joint.

## Conclusions

This case underscores the importance of maintaining a high index of suspicion for rare presentations of osteomyelitis, particularly in atypical locations such as the capitellum. While septic arthritis and osteomyelitis are well-documented entities, the presentation of spontaneous capitellum osteomyelitis in a previously healthy patient remains an exceedingly rare phenomenon. The use of POCUS played a critical role in the timely identification of the joint effusion, and ultrasound-guided arthrocentesis, allowing for early identification and management of a potentially serious condition. Despite initial findings suggestive of septic arthritis, negative synovial and blood cultures highlighted the diagnostic complexity of this case, ultimately resolved with MRI imaging. This emphasizes the value of advanced imaging, such as MRI, in cases where initial diagnostics are inconclusive or when traditional clinical and laboratory findings fail to reveal an infectious source. Early identification and treatment of osteomyelitis and septic arthritis are crucial to prevent serious complications including long-term functional impairment, limb deformity, or systemic sequelae. 
